# Parents of Urban Adolescents in Harlem, New York, and the Internet: A Cross-sectional Survey on Preferred Resources for Health Information

**DOI:** 10.2196/jmir.6.4.e43

**Published:** 2004-12-03

**Authors:** Alwyn T Cohall, Renee Cohall, Bonnie Dye, Sheila Dini, Roger D Vaughan

**Affiliations:** ^3^Department of BiostatisticsMailman School of Public HealthColumbia UniversityNew York NYUSA; ^2^Department of Population and Family HealthMailman School of Public HealthColumbia UniversityNew York NYUSA; ^1^Harlem Health Promotion CenterDepartment of Sociomedical SciencesMailman School of Public HealthColumbia UniversityNew York NYUSA

**Keywords:** Internet, urban, minority, parents, adolescents, health, school

## Abstract

**Background:**

Vulnerable populations suffer disproportionately from a variety of health conditions. Access to health information is an important component of health promotion. Reports suggest that while health providers and print media are traditional sources of information, the Internet may be becoming an increasingly important resource for consumers. Particularly, for parents of urban adolescents of color, the Internet could prove to be a valuable asset in helping them understand adolescent health and behavioral issues.

**Objective:**

To determine the types of adolescent health and behavioral issues of concern to parents of color and to assess their preference for sources of health information, including the Internet.

**Methods:**

A confidential, self-administered survey was administered to parents (largely of African American and Hispanic descent) of 9th-grade students over a 2-year period during 2001-2002 in Harlem, NY. The instrument assessed health and behavioral topics of concern, preferred resources for information, ownership and utilization patterns of computers and the Internet, and interest in obtaining additional computer/Internet training.

**Results:**

A total of 419 surveys were completed; 165 in 2001 (67% response rate) and 254 in 2002 (no response rate available). Analysis of responses indicated a substantial degree of interest in obtaining information about a variety of adolescent health issues, including: HIV, sexually transmitted infections, , mental health concerns and relationships with family and peers. While home ownership of computers (84%) and access to the Internet were reasonably high (74%), use of the Internet for health information was low (14%). However, 62% of parents indicated a strong desire to have more instruction on computers and the Internet.

**Conclusions:**

Compared to other sources of health information, the Internet is underutilized by urban parents of color. Additional research is needed to identify strategies to improve utilization and assess subsequent impact on parenting activities.

## Introduction

When properly educated, motivated and supported, parents can be instrumental in providing better monitoring of their adolescent's activities with resultant reductions in risk-taking behaviors [1]. Parents, in general, desire more information and guidance on child rearing [2], and health providers can play an important role in educating parents and/or steering them towards additional sources of information on health and behavioral issues. However, while the average duration of pediatric well-child visits has increased to about 15 minutes [3], only 15%-20% of parents recall their child's provider discussing psychosocial issues with them [2].

Parents of adolescents are at an even greater disadvantage than parents of younger children, since adolescents have fewer routine visits than infants and children [4]. Additionally, to ensure confidentiality, parents may appropriately be asked to wait in the waiting room during at least part of the encounter and, in some cases, parents may not even accompany their adolescent to office visits. Thus, parents of adolescents may have less contact with health providers than parents of younger children.

To supplement advice given by health providers, parents may turn to popular magazines. While several exist for parents of infants and younger children, few have regular features or special sections that cover the 13-19 age group, and no magazine exists exclusively for parents of adolescents. Finally, books are important information resources for parents, but a recent review by 1 of the authors in this study found that of the over 3,000 titles carried by Amazon.com on child health, only about 25% focused exclusively on adolescents (unpublished observations).

Over the last several years, surveys suggest that millions of Americans are increasingly turning to the Internet, not only for communication and entertainment, but for health information as well. Available information reveals that as of March 2003, 66% of Internet users have accessed the Web for health or medical information [5]. Having children in the home is a particular impetus for having both computer and Internet access. Allen and Rainie reported that among adults who do not currently own a computer, parents are more inclined to “intend to purchase a computer and log on” in the near future, than non-parents [6]. In addition to educational enrichment, parents are particularly interested in using the Internet to seek out health information with respect to their children [6].

Although inequities in socioeconomic status and education contribute to disparities in access to the Internet (the “Digital Divide”), evidence suggests that the gap may be narrowing. In particular, the percentage of African American and Hispanics/Latinos logging on to the Internet is rising faster than among whites [7]. Additionally, there is a suggestion that African Americans are using the Internet in increasing numbers for health information, because they have been “cut off” from traditional forms of health information [8]. For example, only 3% of the books available on Amazon.com deal specifically with minority youth issues. Thus, while it is important that all parents obtain needed resources to assist them in coping with the rigors of parenting, parents of color are particularly in need. In general, youth of color are over-represented with respect to many health-compromising behaviors. For example, the 2003 Youth Risk Behavior Surveillance reports a higher prevalence of sexual intercourse (67.3%, 51.4%, 41.8%), pregnancy (9.1%, 6.4%, 2.3%), and attempted suicide (8.4%, 10.6%, 6.9%) among African American and Hispanic youth as compared to their white peers respectively [9].

Potentially, the Internet could mitigate some of the gaps in health information for parents of color; however, little is known about their preferences for health information resources and, specifically their use of the Internet to seek information about adolescent health and behavioral topics. This study was designed to obtain more information on those issues, as part of a larger project (ie, the School Health Promotion Initiative) developed by the Harlem Health Promotion Center. This center is 1 of 33 prevention research centers funded by the Centers for Disease Control and Prevention to engage community partners such as government agencies, schools, and community-based organizations in the design and implementation of applied prevention research programs. The goal of the School Health Promotion Initiative is to enhance opportunities for high school students to engage in health promotion activities. A related area of investigation centers on the crucial role parents play in enhancing adolescent health promotion. This paper focuses on the latter component of the overall initiative.

Specifically, we are interested in learning more about parental preferences for sources of health information for themselves and their children, parental access to computers and the Internet, and ultimately, parental interest in using the Internet to obtain health information.

## Methods

The study was conducted in a public high school in Harlem, New York where parents (or guardians) of incoming 9th-grade students participated in a 1-day orientation session prior to the start of the academic year. During the 9th-grade orientation sessions in June 2001 and 2002, an anonymous 24-item questionnaire, offered in both English and Spanish, was administered (see Multimedia Appendix for the questionnaire). Inclusion criteria for consideration in the study were being a parent or guardian of a child entering the 9th grade of the school in the fall following the orientation. The project was reviewed and approved by the Institutional Review Board of Columbia University and by the Office of External Research for the New York City Board of Education. All participants were informed that their participation was voluntary and that any information obtained would be kept confidential. The questionnaire collected data on: parental demographics, insurance coverage, usual source of health care, and frequency of visits, for their adolescents. Further, they were encouraged to prioritize their preferred sources of health information, and provide information on their use of computers and the Internet. Finally, their interest in obtaining training on computers and the Internet was assessed.

Surveys were collected and entered with a less than 1% data entry error rate. SPSS version 11.0 was used for record keeping and subsequent analysis. Frequencies were calculated and grouped by general themes, including demographics, health care visits, and sources of information on health issues. Chi-square tests were used to examine relationships where the dependant variable was categorical or ordinal. If the dependant variable was continuous, a t-test was used. Only differences that were significant at a *P* < .05 level are reported.

## Results

### Demographic Characteristics of Respondents

A total of 419 surveys were completed; 165 in 2001, and 254 in 2002. Data did not differ significantly on any variables based on the year of collection. During the first collection year, out of a total of 367 parents of entering 9th-grade students, 248 (68%) attended the school orientation program and 165 returned a completed survey (67% of attendees, or 45% of the overall sample frame). Although year 2 participation rates were not available, there is little reason to believe that response rates would be dissimilar. Additionally, there were no significant differences in the demographic profile of the sample between 2001 and 2002, nor were responses significantly different between the 2 years; therefore data sets for both years were combined for analysis.

**Table 1:** Demographic profile (N = 419)

	**n**	**%**
**Age**		
Less than 29	8	3
30-39	132	42
40-49	136	43
50 and older	39	12
Unknown/no response	104	
**Race/Ethnicity**		
Black/African American	177	47
Hispanic/Latino	175	47
Other*	24	6
Unknown/no response	43	
**Gender**		
Female	325	86
Male	51	14
Unknown/no response	43	
**Relationship to Child**		
Parent	353	92
Grandparent	7	2
Aunt/uncle	11	3
Other	11	3
Unknown/no response	37	
**Health Insurance**		
None	21	5
Medicaid	135	34
Child Health Plus	52	13
Private insurance	189	48
Unknown/no response	22	

^*^ Includes white, Native American, Asian and other.

Since 92% of the sample consisted of parents (n = 353), for this analysis we will use the term parent to refer to all participants, including guardians. Eighty-six percent of our sample were women (n = 325). Forty-seven percent of the sample identified themselves as black or African American (n = 177); a like number (47%) self-identified as Hispanic (n = 175), and 6% as white, Native American, Asian or other (n = 24). Because only 1 individual was identified as white, we did not exclude this person from our analysis since our paper focused on urban parents rather than exclusively on people of color and 1 person out of 419 would not appreciably alter the data. Most participants (85%) were 30-49 years old (n = 268). Although specific data on socioeconomic status is not available, 46.4% of adolescents in the school qualify for free lunch, a proxy for low socioeconomic status. Table 1 illustrates the demographic profile of the sample.

### Parents' Sources of Health Information

Parents were asked a series of questions regarding health information such as: where they believed their children received health information, where they *preferred* their child receive health information, and where they themselves received health information. Table 2 summarizes their responses. Most respondents believed that family (n = 252; 66%) and school (n = 154; 40%) were the main sources of health information for adolescents. By contrast, in terms of *preferred sources* of health information, support provided by health professionals (n = 240; 62%) equaled family members (n = 240; 62%). In terms of their own sources for health information, health professionals (n = 213; 57%) held the most prominent ranking, followed by magazines and newspapers (n = 173; 46%), television (n = 100; 27%) and family members (n = 102; 27%).

**Table 2:** Current sources of health information for parents and adolescents*

	My child gets information on health issues from…		I would prefer that my child get information on health issues from…		I get information on health issues from…	
	n	%	n	%	n	%
Church	27	7	30	8	28	7
Family member	252	66	240	62	102	27
Friends	96	25	8	2	40	11
Healthcare provider	113	29	240	62	213	57
Internet	34	9	27	7	53	14
Magazines/newspapers	94	25	51	13	173	46
Other	25	7	20	5	44	12
School	154	40	200	52	69	18
TV	113	29	28	7	100	27

^*^ Respondents were asked to circle all that apply. Hence, results do not sum to 100%.

**Table 3:** Profile of Internet use by parents (N = 419)*

	**Yes** **n**	**Yes** **%**
**Would you be interested in attending a workshop designed to help parents learn how to better use the Internet?**	216	62
**Do you have Internet access at home?**	286	74
**Do you have Internet access at work?**	121	35†
		
**How often do you use the Internet?**		
never	100	26
less than once a month	43	11
1-3 times per month	49	13
1-3 times per week	101	27
Everyday	88	23
Unknown/no response	38	

^*^ n's for selected characteristics may vary due to missing data.

^†^ Question asked “Do you have Internet access at work?” but did not allow for those who were unemployed. As such, 18% (n = 75) of the responses were missing.

### Profile of Internet Access and Utilization

Eighty-four percent of participants indicated that they had a computer at home (n = 325). Seventy-four percent had access to the Internet at home (n = 286) and 35% had access at work (n = 121). However, while 23% indicated that they used the Internet everyday (n = 88), and 27% used it 1 to 3 times weekly (n = 101), 26% indicated they never used the Internet (n = 100) (See Table 3).

Access to the Internet (either at home or work) did not differ based on age or gender; however, African Americans (66%) were nearly twice as likely to have access to the Internet at work as Latinos (34%) (Pearson's χ^2^ = 19.89, *P* < .001). Younger parents (under the age of 40) were slightly more likely to use the Internet (81%) than those parents over the age of 40 (70%) (Pearson's χ^2^ = 4.8, *P* = .03).

Health information seeking via the Internet was relatively low. Fourteen percent of parents used the Internet as a health information resource (n = 53), and 9% thought their children were getting health information on the Internet (n = 34). Only 7% *preferred* the Internet to be a source for health information for their adolescents (n = 27) (Table 2).

While there were no significant differences in responses based on age or gender, among those who used the Internet for health information, African Americans (68%) were more than twice as likely as Latinos (32%) to identify the Internet as a source of health information (Pearson's χ^2^ = 7.23, *P* = .007).

### Interest in Attending Workshops in School

Although the percentage of regular Internet users was low, 62% of respondents indicated they would be interested in attending a workshop on how to use the Internet (n = 216) (Table 3). Not surprisingly, parents who identified that they never used the Internet were more likely to express interest in attending a workshop to improve their Internet skills (75% of non-users versus 60% of users). This interest indicates that it is not lack of interest, but rather lack of familiarity with the Internet that is hindering some parents from using the Internet. Further, while traditional sources of health communication (eg, newsletters, health providers, small group workshops) were cited as preferred means of obtaining health information, 27% reported that they would like to receive information on adolescent health issues via the Internet (Table 4).

**Table 4:** Preferred channels for future receipt of health information by parents*

**Which of the following ways would you like to receive information on teen health issues? (Choose at least 2.)**		
	n	%
Monthly newsletter	257	69
Health provider	162	43
Workshops for parents to be held at school	157	42
Internet website for parents	102	27
Health videos	77	21
Interactive CD-ROM	37	10
Other	4	1

^*^ Respondents were asked to circle all that apply. Hence, results do not sum to 100%.

## Discussion

### Limitations

Before the implications of these results are presented, there are several limitations of this study that should be addressed. First, the internal and external validity of these results may be limited. Only 68% of the parents of 9th graders attended the orientation, and only 67% of those returned the survey for an overall participation rate of 46% for the 2001 survey. The participation rate for 2002 is unknown, but assumed similar. As such, these results may not reflect the knowledge and attitudes of all parents of 9th graders in this school. Additionally, since our sample consisted primarily of minority parents in an urban community, these results may not necessarily be generalizable to non-minority parents or to parents in rural or suburban settings. Additionally, these results were obtained 2-3 years ago and may understate current trends in access and utilization of the Internet.

### Implications

Nonetheless, several interesting findings can be noted. The majority of parents in our sample reported home ownership of computers (n = 325; 84%) and Internet access (n = 286; 74%). Half of the participants reported using the Internet on at least a weekly basis at home or at work (n = 189). This is substantially higher than national samples of people of color. For example, a Pew Study of the Internet and American Life conducted in 2002 found that 45% of African American and 54% of English-speaking Hispanics reported access to the Internet [7]. It has been noted, however, that parental status, younger age and urban residence are positively correlated with Internet access [7], which may provide partial explanation for the discrepancy between our sample of primarily young urban parents and national surveys which sampled a broader cross section of the population. For example, results from a 2003 Pew Internet and American Life report indicated that parents in general were more likely to report Internet use than non-parents (75% of parents reported usage versus 57% of non-parents) [10]. However, 26% of our total sample reported never accessing the Internet (n = 100).

Although we did not ask specific follow-up questions, based on our experience and review of the literature, reasons for non-use may be varied. In the past, cost may have been a notable deterrent, but recent advances in technology have dramatically lowered the costs of a basic computer and Internet set-up. As noted, the majority of our sample had access at home. However, access need not necessarily be equated with utilization. Stanley, in her study of computer utilization by residents of a low-income community in California, suggests that psychosocial issues may play a more significant role in creating and maintaining barriers for computer and Internet use among adults in low-income communities [11]. Lenhart concurs, noting that some individuals fail to see the relevance of computers and the Internet, are embarrassed or intimidated by technology, or are concerned about fraud or disturbing content [7]. Anecdotal conversations with parents in our community reveal similar misgivings. However, additional research is necessary to delineate the particular factors hampering utilization of the Internet by parents in this sample.

With respect to health information seeking, similar to other reports of minority adults [12], parents in our sample cited health professionals as their preferred source of health information. Other important resources include magazines and newspapers. Adults in our sample reported unexpectedly low rates of health information seeking via the Internet. Only 14% reported seeking health information on the Internet. Overall, parents rated the Internet 6th on a list of 9 sources of health information.

Reasons for low utilization may be varied. As noted above, psychosocial issues may hamper access and utilization. Further, parents with limited Internet skills may lack the ability to accurately search and retrieve valuable health information on the Web. Additionally, even skilled parents may become frustrated in finding easy-to-understand information. A survey of pediatric patient information materials on the Web revealed many are written at a 12th-grade reading level. Current recommendations suggest development of materials at the 8th-grade level or lower [13]. Concerns about lack of available content, and cultural relevance have been cited as common complaints by minority adults [14]. Finally, language may be a barrier. Latino parents in our sample were less likely to access health information on the Internet, as compared to African American parents. The dearth of Spanish-language content may be an impediment to Latino parents finding and retrieving information online [13].

Further, only 9% of parents believed their children were getting information from the Internet. By contrast, both national and local studies indicate that adolescents of color are turning to the Internet in significant numbers to seek health information. The Kaiser Family Foundation Generation Rx.com survey revealed that 75% of adolescents sought health information on the Internet. Similarly, surveys conducted in low-income communities in New York City suggest that 55 % of minority adolescents have obtained health information on the Web [15].

The discrepancy between what parents believe youth are doing on the Internet and what actually is occurring may reflect a general lack of parental awareness of how interested youth are about health issues. Additionally, it may illustrate a lack of parental monitoring of adolescent Internet activities.

Further, only 7% of parents preferred the Internet as a source of information about health for their adolescents. This may suggest parental concern about the potential for youth accessing pornographic information, as well as the potential for youth being subject to untoward advances from sexual predators [7]. It will be important to elucidate and address parental concerns as they may potentially hamper adolescent utilization of the Internet for health promotion.

Of note, despite their reservations, 27% of parents indicated they would be interested in receiving information on adolescent health issues delivered by an Internet website (n = 102). Health providers [16], may be of assistance by identifying user-friendly, peer-reviewed, medically accurate Web resources for parents.

Additionally, 62% expressed interest in attending workshops to obtain or improve their computer/Internet literacy (n = 216). Community technology centers (CTCs) exist in many vulnerable communities, and provide free or low-cost access and training. Particular for novice users, CTCs “play a pivotal role in helping (new computer users) overcome their resistances…the majority of new computer users quickly overcame their fears and reservations once they had an actual hands-on computer encounter in a supportive and comfortable adult learning environment.” However, “many individuals do not know that CTCs exist or about the services they offer…” [11]. Therefore, a key role for health providers and local schools may be to identify CTCs in their communities and suggest parents access their services to improve their computer/Internet literacy.

Additionally, over the past several years, a significant upgrade in the technological capacity of schools has been undertaken. Many not only have the computer resources and Internet access to provide educational advancement for students, but basic training for parents, as well, during off-hours. As such, schools may provide a valuable service for parents in vulnerable communities by also providing basic instruction. This may be increasingly important, as many schools are developing websites wherein information about school activities and their child's academic performance can be relayed to parents. Additionally, the potential also exists to improve parent-teacher communication via email. However, if parents lack the skills or the ability to access the Internet, this potential will not be easily realized.

Finally, the potential for adolescents to influence parental behavior should be considered. In addition to being avid seekers of health information themselves, 39% of all adolescents (52% of African American adolescents and 42% of Hispanic adolescents) report changing their behavior because of online content. Fifty-three percent of adolescents say they have had a conversation with a parent or other adult about something they had seen online [17]. Adolescents are the most technologically proficient members of most households, and as such, may be able to teach those parents who are novice users how to more efficiently access information. In a quarter of families with Internet access, teens often master the technology before their parents and take the lead on teaching them [6]. Thus, adolescents may indirectly (through modeling) or directly (through teaching) influence parents to become Internet health seekers.

However, this study suggests that additional research is warranted to further elucidate factors that may enhance utilization of the Internet for health information. Additionally, if utilized, the comparative degree of satisfaction with Internet resources as compared to more traditional forms of health information would be important to ascertain. The impact of Internet-based health information on parental awareness of adolescent health and behavioral issues is currently unknown. Potentially, a positive feedback loop could be envisioned by which adolescent health promotion is enhanced, not only by the adolescent's own efforts but also by reinforcement coming from better-informed parents.

### Conclusion

Our study indicates that parents of adolescents in an urban community are interested in receiving additional information and support to become more knowledgeable and informed. At present, the Internet is potentially an important but underutilized tool in their arsenal. Parents in urban communities may need specific guidance and support in order to take better advantage of this valuable resource. Creation of culturally relevant content of appropriate literacy levels will be important to maintain the interest and attention of parents of color. Further, increased efforts to provide Spanish-language content may be an important factor in making the Internet accessible to Latino parents.

## Multimedia Appendix

### Survey Instrument

24 item survey instrument fielded in 2000 and 2001.
