# Supplementary material for: Parents of Urban Adolescents in Harlem, New York, and the Internet: A Cross-sectional Survey on Preferred Resources for Health Information
Source: J Med Internet Res. 2004 Dec 3;6(4):e43. doi: 10.2196/jmir.6.4.e43 (PMC1550623; doi:10.2196/jmir.6.4.e43)
Supplement: Supplementary file 1 [file jmir_v6i4e43_app1.pdf]

ID# __ __ __

Dear Parents/Guardians: Thank you for filling out the School Health Promotion Initiative's Parent Survey. The answers you give below are confidential and anonymous. You participation is voluntary, but will help us better understand where you and your child currently receive health information and how we can help provide you with additional resources. The following questions apply to the child in your family who will be attending 9th grade in this school starting September of 2002.

1. Where does your child usually go when he or she is sick or needs advice about health? (circle all that apply)
   ① Clinic in a hospital
   ② Health center or clinic not in a hospital
   ③ Doctor's office that is not part of a clinic or hospital
   ④ Emergency room
   ② Other (specify: ____________)

2. What kind of health provider usually takes care of your child?
   ① Pediatrician
   ② Adolescent medicine doctor
   ③ Internist
   ④ Gynecologist
   ⑤ Other(specify: ____________)

3. What type of health insurance does your son or daughter have?
   ① None
   ② Medicaid
   ③ Child Health Plus
   ④ Private insurance (Oxford, HIP, GHI etc.)

4. When was the last time your son or daughter had a complete health check-up?
   _ _ / _ _
   month/year

5. Do you usually accompany your child for health visits?
   ① Yes
   ② No

6. During their last visit, at <u>any</u> time, did the health provider visit, did to the health provider speak privately to your child?
   ① Yes
   ② No
   ③ Not sure

7. How important is it **to you** for your child's health provider to talk to your child about each of the following topics?

	Very Important	Somewhat Important	Not Important	Not Sure If Important	Do Not Discuss
Abuse (physical, sexual, verbal)	①	②	③	④	⑤
AIDS	①	②	③	④	⑤
Alcohol/Drug Abuse	①	②	③	④	⑤
Behavior problems/ discipline	①	②	③	④	⑤
Body Image/ Self Esteem	①	②	③	④	⑤
Contraception	①	②	③	④	⑤
Depression	①	②	③	④	⑤
Eating Problems	①	②	③	④	⑤
Exercise/Sports	①	②	③	④	⑤
Family Illness	①	②	③	④	⑤
Family Relationships	①	②	③	④	⑤
Friends/Peer Relationships	①	②	③	④	⑤
Growth	①	②	③	④	⑤
Hygiene	①	②	③	④	⑤
Nutrition	①	②	③	④	⑤
Pregnancy	①	②	③	④	⑤
"Safer" sex	①	②	③	④	⑤
Safety (seat belt, helmets)	①	②	③	④	⑤
School issues	①	②	③	④	⑤
Self-exam (Breast or Testicular)	①	②	③	④	⑤
Sexual Development	①	②	③	④	⑤
Sexually Transmitted Diseases	①	②	③	④	⑤
Skin care	①	②	③	④	⑤
Smoking	①	②	③	④	⑤
Stress	①	②	③	④	⑤
Suicide	①	②	③	④	⑤

8. Do you know if the health provider discussed or provided information to your child on any of these topics during the last visit?
   ① Yes ➔ Which ones? (specify: ____________)
   ② No

OVER ➔

9. Which of the above items in question 7, are the most **important** topics for **you** to receive more information on so that **you can help your child?**

① ______________________
② ______________________
③ ______________________

10. Where do you think your child usually gets information on health issues? (choose at least two)

① Friends
② Magazines/newspapers
③ TV
④ Family member(specify: ____________)
⑤ Internet
⑥ Healthcare provider
⑦ Church
⑧ School
⑨ Other(specify: ____________________)

11. Where would you **prefer** that your child get their information on health issues? (choose at least two)

① Friends
② Magazines/newspapers
③ TV
④ Family member(specify: ____________)
⑤ Internet
⑥ Healthcare provider
⑦ Church
⑧ School
⑨ Other(specify: ___________________)

12. Where do **you** usually get your information on **adolescent** health issues, in general? (choose at least two)

① Friends
② Magazines/newspapers
③ TV
④ Family member(specify: ____________)
⑤ Internet
⑥ Healthcare provider
⑦ Church
⑧ School
⑨ Other(specify: ___________________)

13. Which of the following ways would **you** like to receive information on teen health issues? (choose at least two)

① Monthly newsletter
② Internet website for parents
③ Workshops for parents to be held at school
④ Interactive CD-ROM
⑤ Health provider
⑥ Health videos
⑦ Other(specify: ___________________)

14. When would be the best time for you to attend a health information workshop at your school?

① Mon-Fri. (9-5pm)
② Mon-Fri. (7-9pm)
③ Saturday (10-12pm)
④ Saturday (2-4 pm)
⑤ Other(specify: ____________)

15. Do you have a computer at home?

① Yes ② No

16. Do you have Internet access at home?

① Yes ② No

17. Do you have Internet access at work?

① Yes ② No

18. How often do you use the Internet?

① Never
② Less than once a month
③ 1-3 times per month
④ 1-3 times per week
⑤ Everyday

19. Would you be interested in attending a workshop designed to help parents learn how to better use the Internet?

① Yes ② No

If yes, what are the best times for you?________

20. How old are you? ____

21. Are you male or female?

① Female ② Male

22. What is your race or ethnicity?

① Black/African American
② White
③ Hispanic/Latino
④ Native American
⑤ Asian
⑦ Other

23. What is your relationship to the student attending this school in the fall?

① Parent (e.g. mother or father) or step-parent
② Step-parent
③ Grandparent
④ Aunt/Uncle
⑤ Legal guardian, not blood relative
⑥ Foster parent
⑦ Other (specify: ________)

24. Do you have any other children under 18 at home?

① Yes ② No

If yes, do any attend this school?

① Yes ② No

Thank you for your participation. Please return this form to Renee or Jennifer.
If you have any questions after we leave, you may reach us at our offices, (###) ###-####.
